# Measurement of volatile organic compounds emitted in libraries and archives: an inferential indicator of paper decay?

**DOI:** 10.1186/1752-153X-6-42

**Published:** 2012-05-15

**Authors:** Lorraine T Gibson, Abdunaser Ewlad-Ahmed, Barry Knight, Velson Horie, Gemma Mitchell, Claire J Robertson

**Affiliations:** 1 Department of Pure and Applied Chemistry, WestCHEM, University of Strathclyde, Glasgow, G1 1XL, UK; 2 The British Library, St. Pancras, 96 Euston Road, London, NW1 2DB, UK

**Keywords:** Indoor air monitoring, Passive sampling, Active sampling, Tenax TA, Paper degradation, Library conservation

## Abstract

**Background:**

A sampling campaign of indoor air was conducted to assess the typical concentration of indoor air pollutants in 8 National Libraries and Archives across the U.K. and Ireland. At each site, two locations were chosen that contained various objects in the collection (paper, parchment, microfilm, photographic material etc.) and one location was chosen to act as a sampling reference location (placed in a corridor or entrance hallway).

**Results:**

Of the locations surveyed, no measurable levels of sulfur dioxide were detected and low formaldehyde vapour (< 18 μg m^-3^) was measured throughout. Acetic and formic acids were measured in all locations with, for the most part, higher acetic acid levels in areas with objects compared to reference locations. A large variety of volatile organic compounds (VOCs) was measured in all locations, in variable concentrations, however furfural was the only VOC to be identified consistently at higher concentration in locations with paper-based collections, compared to those locations without objects. To cross-reference the sampling data with VOCs emitted directly from books, further studies were conducted to assess emissions from paper using solid phase microextraction (SPME) fibres and a newly developed method of analysis; collection of VOCs onto a polydimethylsiloxane (PDMS) elastomer strip.

**Conclusions:**

In this study acetic acid and furfural levels were consistently higher in concentration when measured in locations which contained paper-based items. It is therefore suggested that both acetic acid and furfural (possibly also trimethylbenzenes, ethyltoluene, decane and camphor) may be present in the indoor atmosphere as a result of cellulose degradation and together may act as an inferential non-invasive marker for the deterioration of paper. Direct VOC sampling was successfully achieved using SPME fibres and analytes found in the indoor air were also identified as emissive by-products from paper. Finally a new non-invasive, method of VOC collection using PDMS strips was shown to be an effective, economical and efficient way of examining VOC emissions directly from the pages of a book and confirmed that toluene, furfural, benzaldehyde, ethylhexanol, nonanal and decanal were the most concentrated VOCs emitted directly from paper measured in this study.

## Background

Access to information contained within books is of prime importance to our academic, social, recreational and artistic development. Books contain knowledge of past centuries and inform us of previous generations and technical developments; providing a wealth of historical data. It is therefore important that books remain in conditions that permit their access and that those which are under threat, from a stability point of view, are identified and restored to ensure the continued longevity of information flow.

It is known that the stability of a paper is, to a large extent, governed by its production. Ancient books can be more stable than modern (19 – 20^th^ c) books as they used linen, hemp or cotton rag in early production processes thus providing high quality cellulosic material. Increased demand for paper from the 19^th^ c onwards led to the use of lower quality cellulosic materials (woodpulp and straw for example) and since the mid-19^th^ century wood has been the principal choice of ingredient. Use of wood in paper manufacturing processes has resulted in the introduction of hemicellulose and lignin, together with other organic and inorganic additives, into the paper product. Thus paper produced from 1850 – 1950 is more acidic (with a pH of around 4–5) and, as the stability of paper is strongly dependent on its pH [1], this has resulted in the production of many books with fragile paper that may not survive after the 22^nd^ century [2]. One of the main routes of paper deterioration is by acid catalysed hydrolysis of cellulose, resulting in depolymerisation (Dp) and loss of structural integrity [3].

Traditional methods of paper analysis therefore focus on measurement of pH [4], mechanical strength [5] and/or an assessment of depolymerisation by size exclusion chromatography [6] or spectroscopy [7-9]. Fourier transform infrared spectroscopy (FTIR) has also been used to assess paper degradation in relation to the presence of metal ions which can catalyse the oxidative degradation of cellulose. Faubel et al [10] used FTIR to assess two different iron gall inks and examine the effect of higher metal ion concentration on the rate of paper degradation. Analytical methods have also been applied to assess paper after artificial aging. In one study [11] conditions of 23°C and 50% RH were used to assess the effect of Cr (II) or Fe (III) ions on degradation kinetics [11]. Indeed transition metals have been shown to catalyse the oxidative degradation of cellulose [12]. Although less commonly applied, nuclear magnetic resonance (NMR) spectroscopy has also been used to study paper degradation after samples of paper were artificially aged by treatment with an oxidant reagent [13]. Unilateral NMR relaxometry was used to examine the size of the water pores in the paper and their influence on the observed degradation.

More recently attention has focussed on the volatile by-products emitted by paper as it deteriorates, in an attempt to provide a truly non-invasive measurement of paper stability. In one study [14], 8 classes of VOCs were emitted from paper and included; carboxylic acids, alcohols, aldehydes, benzenic derivatives, alkyl benzenes, aliphatic hydrocarbons, esters and polycyclic aromatic hydrocarbons. Many of the emitted volatiles, (acetic acid, formic acid, furfural, 4- hydroxybenzoic acid, 4-hydroxyacetophenone, 4-hydroxybenzaldehyde, vanillin), have been attributed to lignin or cellulose degradation [15] and so it may be possible to use these species as chemical deterioration markers. For example, furfural has been linked to paper degradation and has been used as a stability marker in oil immersion applications [3], for historical paper [16] and for the assessment of artificial aging studies on iron gall inks [17]. In some of these initial studies SPME fibres were used to collect the emissive volatiles and it was observed that the quantity, and analyte combination, collected was dependent upon the stationary phase coating used on the fibre [14]. An electronic nose has also been used in paper emission work [18]. Paper samples that had been affected by mould growth were successfully identified and separated from those which had not been affected. However it was concluded that more research was necessary before the electronic nose could come into common use, even though some of the VOCs identified related to compounds which are associated with the ‘smell of books’ [19].

The objective of this study was to further explore research in paper emission by (i) examination of VOC concentrations at selected locations inside national libraries and archives, (ii) assessment of correlations between collected analytes and (iii) determining if volatile markers, emitted directly from books, were measured in collected indoor air samples.

## Results and discussion: Indoor air sampling

### Indoor air sampling locations

Eight partner institutes were involved in the sampling survey (see Table 1), and three sampling locations were chosen at each institute. Two of the locations (referred to as locations ‘A’ or ‘B’) chosen contained paper-based collections, whereas the third location chosen for assessment (location ‘C’) was a sampling reference, ie., the area did not contain any paper-based material. Passive sampling was initiated on day 1 and pollutants were continuously collected for 28 d. Active sampling was conducted on day 1 and a repeat sample was taken onto a replicate Tenax TA tube on day 2. The results given in all tables and figures are the average values for repeat samples.

**Table 1:** Partner institutes and sampling locations

	**Location A**		**Location B**		**Location C**	
**National Archives of Scotland (NAS)**	Thomas Thomson House GC 1		General Register House Room 45 Collection GD45 (GRH)		Thomas Thomson Front Hallway	
**National Library of Scotland (NLS)**	234/D1 Shelf 2/37		10/36- D1 Shelf No10.66		Hallway next to Lift 1111/D1	
	46% RH	17°C	47% RH	19°C	34% RH	21°C
**British Library (BL)**	SCP Basement 3, Comp 1		Colindale D f/4		1^st^ Floor Above Entrance Hallway	
**Cambridge University Library (CUL)**	Map Department Ground Floor F12		Tower 14 Case 11		Legal Deposit Corridor	
**The National Archives (TNA)**	Repository 5B Shelf 237		Repository 6A By Shelves 734-738		3^rd^ Floor Landing Outside Repository 8	
	50% RH	18°C	50% RH	18°C	50% RH	18°C
**Trinity College Dublin (TCD)**	Upper Colonnades Shelf M11		Long Room, Gallery Southside II.37		West Pav (Outside Henry Jones Room)	
**National Libraries of Wales (NLW)**	2003 MB		XL 333 W17- XL 361		Conservation Building Entrance Hallway	
	8851–9764		G Floor			
	STORFA 2 75					
**Bodleian Library Oxford (OULS)**	D Floor Bookstack –rows 216–226 (end of row) Area 2041.88 m^3^		J Floor Bookstack – rows 1575–1590 (end of row) Area 6658.20 m^3^		Ground Floor Corridor (outside Rm 132 Level H)	
	57% RH	22°C	56% RH	20°C	47% RH	22°C

Each location included in the survey differed in the type of building housing the collection, the air handling systems used, as well as the items and fabrication inside the selected sampling sites. Some locations were known to hold approximately a quarter of a million documents consisting of different types of material in addition to paper (microfilm, parchment, photographic material etc.). An indication of the average relative humidity and temperatures, where measured, during sampling is given in Table 1. It was necessary to ensure that locations sampled could be accessed by staff during the sampling period and as an indication of the number of times a room was accessed, locations A and B were monitored at the National Library of Scotland. One door into location A was opened up to 135 times and two of the doors into location B were opened 77 and 90 times during sampling.

### Direct sampling of paper using SPME fibre or elastomer strips

VOCs emitted directly from the pages of a number of books were trapped onto SPME fibres coated with divinylbenzene/carboxen/PDMS or onto small PDMS strips. Prior to use SPME fibres were conditioned by placing them into a gas chromatograph (GC) injection port held at 270°C for 3 h. The PDMS material was used as supplied however it was cut into small strips of 40 mm × 45 mm. During sampling the fibres and PDMS strips were carefully positioned between the pages of the books. Initially sampling was performed for 4 books manufactured between 1907 and 1952 (see Table 2); fibres or strips remained in position for 2 or 5 d, respectively. To ease pressure on the fibres they were removed from the fibre holder before being placed in the book. In addition 8 PDMS strips were placed in between 2 sheets of pages within a book and placed at different locations to determine whether it was possible to look for different furfural emissions across a page. Three books of different age were examined as indicated in Table 3. The SPME fibres and PDMS collection strips were also sent to 7 of the partner institutes to sample the VOCs emitting from a book that was common to each institution. The selected book was The Whitaker’s Almanack – An almanac for the year of our Lord 1903 (J. Whitaker & Sons, London, 1903). Samples were carefully placed between 2 pages, around the middle of each book, for approximately 14 d. Temperature and relative humidity values at each site were in the range 18 – 21°C and 36.2 – 48.2%. Sampling VOCs from the Whitaker’s Almanack demonstrated the usefulness of PDMS strips in collecting VOCs however they were not as sensitive as SPME fibres. Hence a final study was conducted where PDMS fibres were used to sample VOCs emitted from a range of collection items (dating from the 15th c to the late 20th c) held at the archives of the University of Glasgow. Here the PDMS strips remained at the sampling locations for approximately 5 months.

**Table 2:** Books analysed by SPME fibres and PDMS strips

**Title**	**Author**	**Publication date**	**SPME Fibre location**
The Badminton Library- Racing	Arthur Coventry	1907	Page 215
The Determination of Hydrogen Ions	W. Mansfield Clark	1923	Page 235
Whitaker’s Almanack 1925	Not applicable	1925	Page 525
Whitaker’s Almanack 1952	Not applicable	1952	Page 580

**Table 3:** Examination of furfural emission across a page: special distribution analyses

**Book title**	**Author**	**Year**	**Publisher**
Organic Chemistry	E.E. Turner, Margaret M. Harris	1952	Longmans, Green and Co
Spectrochemical analysis	James D. Ingle, Jr., Stanley R. Crouch	1988	Prentice Hall
Chemistry: The Molecular Nature of Matter and Change	Martin S. Silberberg	2003	McGraw Hill

### Passive sampling results of indoor air survey

All SO_2_ concentrations measured were < 1 μg m^-3^, except for locations B and C at the NLS where a low concentration of 1.38 μg m^-3^ was measured. Formaldehyde concentrations were all lower than 18 μg m^-3^ (see Table 4); in some cases location C had the highest measured values and in this study there appeared to be no relationship between formaldehyde emission and paper-based collections. The average concentrations measured for acetic or formic acid vapours are also given in Table 4. In most cases elevated, or similar, acetic acid vapour concentrations were measured at locations A and B compared to measurements taken at the reference location C. There was only one exception where the concentration of acetic acid vapour was significantly higher at the reference location C; at TCD. The measured acetic acid vapour concentration was always higher than that of formic acid vapour; in sampling reference locations (C) by a factor of approximately 2 and when paper-based materials were present (A or B) by a factor of approximately 3–4.

**Table 4:** Passive sampling survey of library and archives

	**Acetic acid /μg m**^**-3**^**Location**			**Formic acid / μg m**^**-3**^**Location**			**Formaldehyde / μg m**^**-3**^**Location**		
	**A**	**B**	**C**	**A**	**B**	**C**	**A**	**B**	**C**
**National Archives of Scotland (NAS)**	250 ± 212	119 ± 16	69 ± 1	58 ± 13	37 ± 12	52 ± 17	6 ± 1	4 ± 1	7 ± 1
**National Library of Scotland (NLS)**	221 ± 195	91 ± 1	98 ± 15	30 ± 6	30 ± 3	42 ± 3	8 ± 1	14 ± 2	12 ± 1
**British Library (BL)**	222 ± 16	348 ± 33	52 ± 27	40 ± 1	310 ± 86	34 ± 13	7 ± 1	15 ± 3	4 ± 1
**Cambridge University Library (CUL)**	158 ± 25	274 ± 83	109 ± 34	67 ± 18	72 ± 21	49 ± 4	18 ± 1	3 ± 1	5 ± 1
**The National Archives (TNA)**	57 ± 30	104 ± 33	36 ± 2	32 ± 4	55 ± 20	64 ± 18	6 ± 1	8 ± 1	8 ± 1
**Trinity College Dublin (TCD)**	195 ± 10	137 ± 72	344 ± 286	56 ± 16	57 ± 6	186 ± 199	12 ± 6	4 ± 1	5 ± 1
**National Libraries of Wales (NLW)**	76 ± 1	108 ± 41	122 ± 15	22 ± 1	30 ± 7	24 ± 2	6 ± 1	7 ± 1	10 ± 1
**Bodleian Library Oxford (OULS)**	123 ± 4	162 ± 21	132 ± 11	48 ± 4	58 ± 24	62 ± 1	5 ± 1	6 ± 1	9 ± 1

### Active sampling results using tenax ta sampling tubes

**Figure 1:**
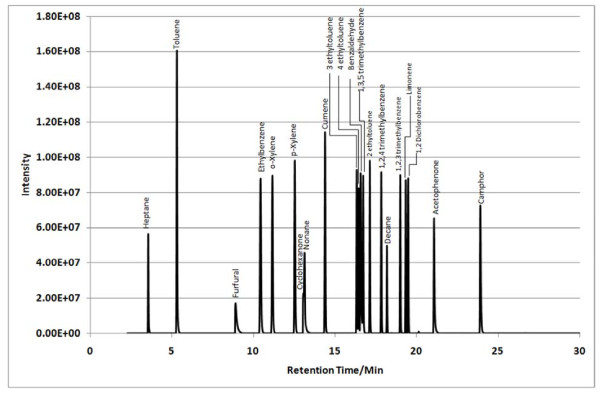
**TIC for a 5 μL injection volume of the 600 ng μL**^**-1**^**standard calibrant solution.**

A 21 analyte calibration standard (analytes were prepared accurately to provide an approximate concentration of 600 ng μL^-1^) was prepared in methanol. A separate GC injection port (Perkin-Elmer Autosystem XL) was used to load known volumes (1–5 μL) of the calibration standard onto a Tenax TA sampling tube using helium as a carrier gas set at a flow of approx 1 cm^3^ s^-1^. The resultant total ion chromatogram (TIC) for the 5 μL calibrant solution is given in Figure 1. A 5 μL volume of 300 ng μL^-1^ of dichloromethane was used as an internal standard and spiked onto calibrant and sampling tubes. Internal standard least squares regression lines were used to correlate analyte masses with peak area and to determine the analyte masses trapped onto sampling tubes during measurement of indoor air locations. Conversion from analyte masses to vapour phase concentrations was achieved by dividing the trapped analyte mass by the volume of air sampled.

**Figure 2:**
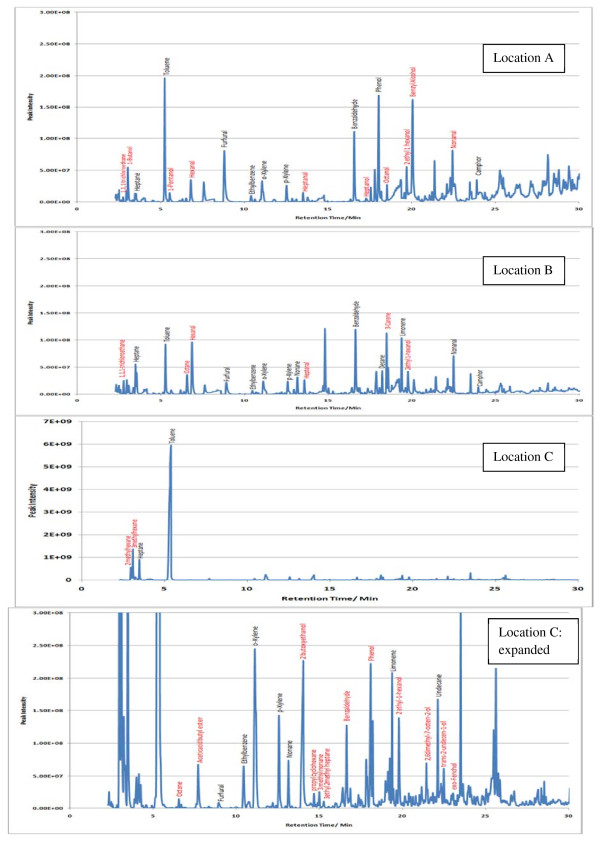
Sample TICs of sampling locations at the National Archives of Scotland.

**Figure 3:**
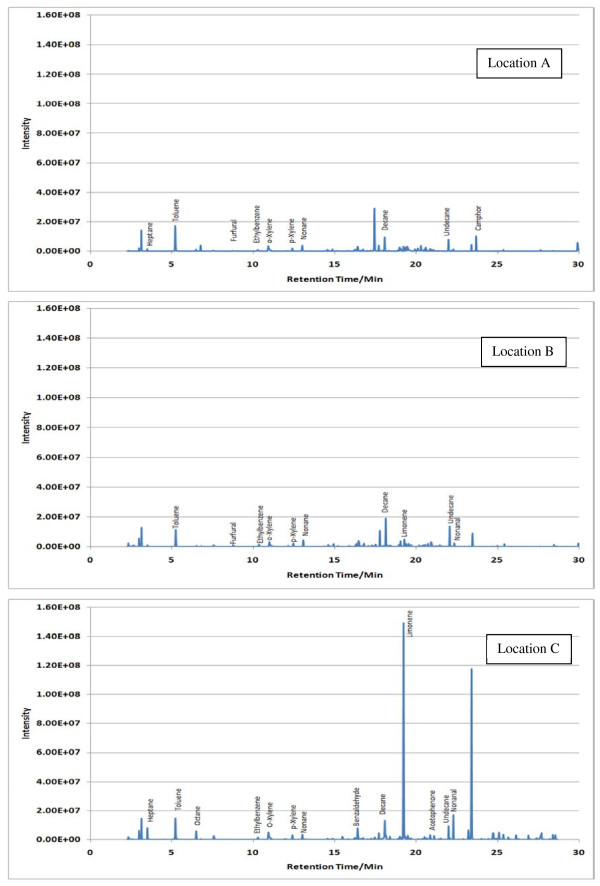
Sample TICs of sampling locations at the National Library of Scotland.

**Figure 4:**
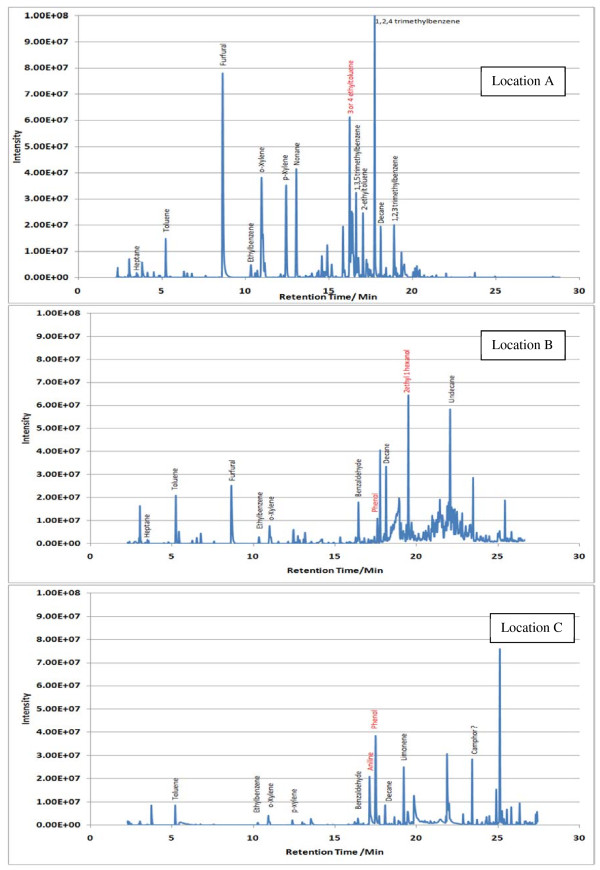
Sample TICs of sampling locations at The British Library.

**Figure 5:**
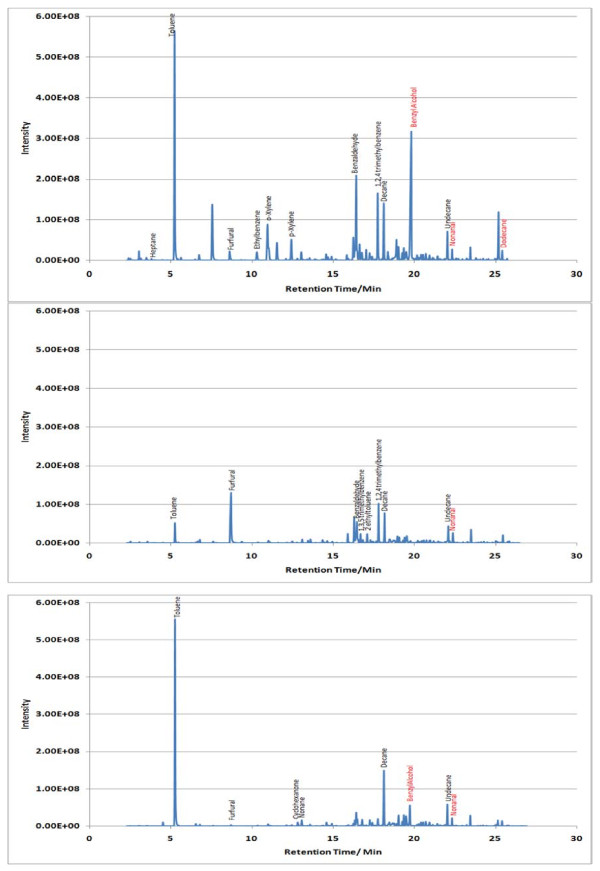
Sample TICs of sampling locations at Cambridge University Libraries.

**Figure 6:**
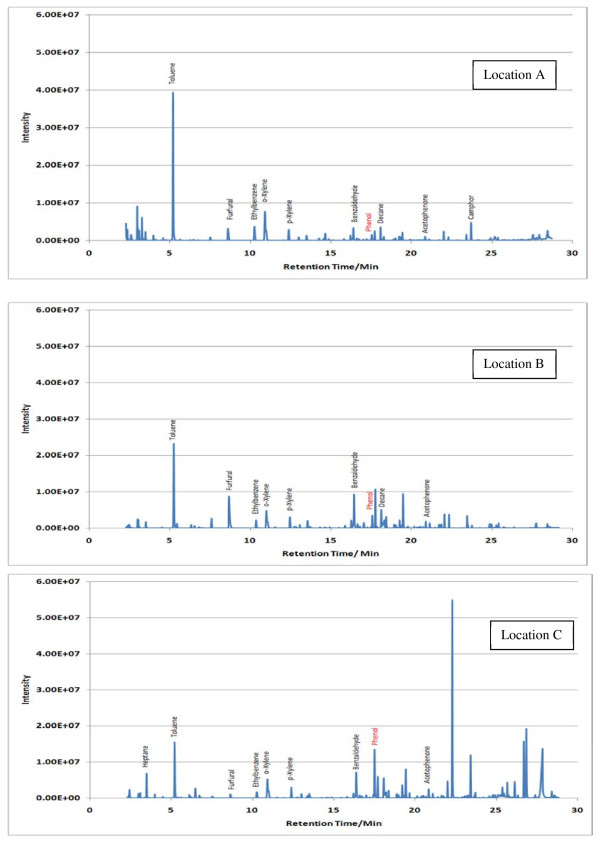
Sample TICs of sampling locations at the National Archives.

**Figure 7:**
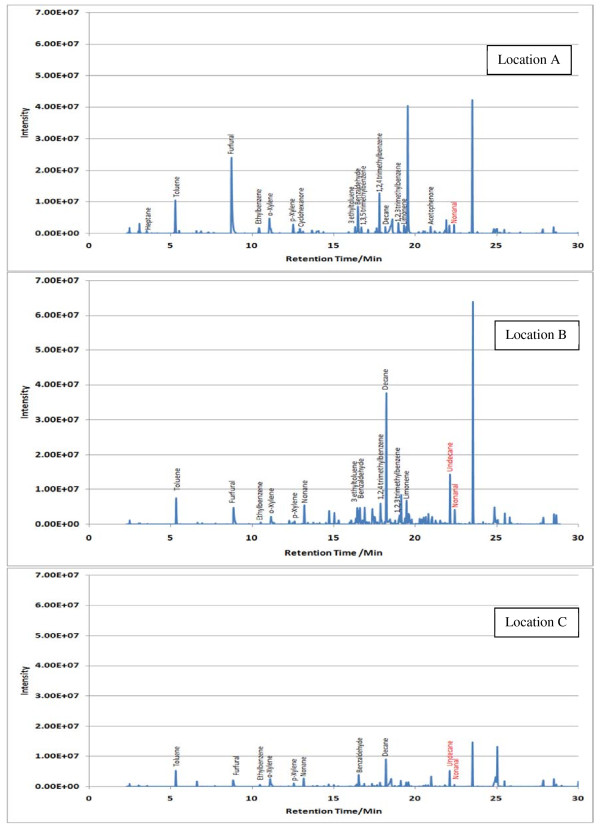
Sample TICs of sampling locations at Trinity College Dublin.

**Figure 8:**
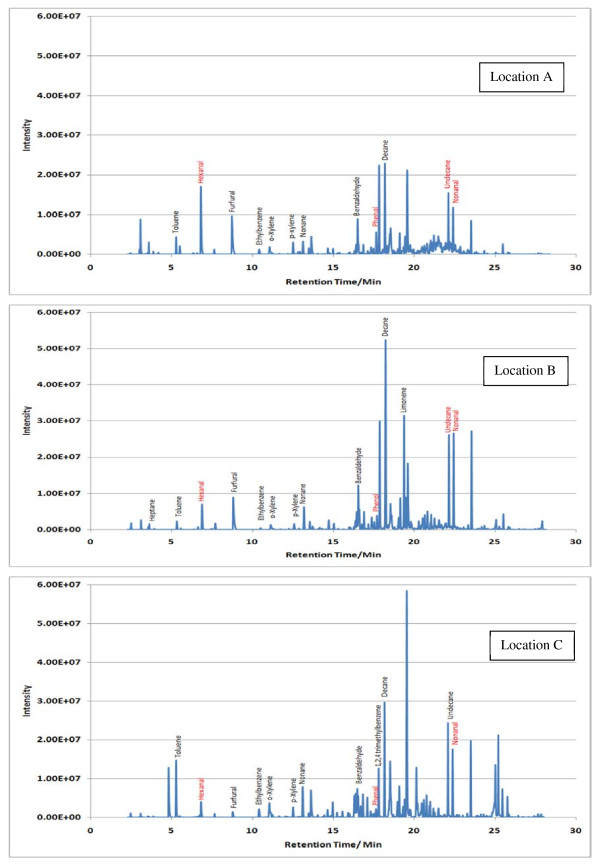
Sample TICs of sampling locations at the National Library of Wales.

**Figure 9:**
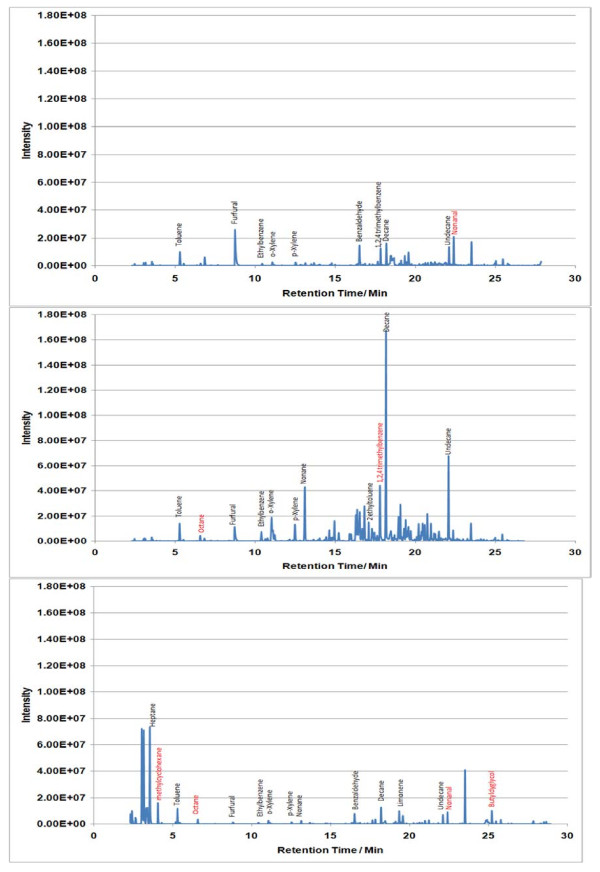
Sample TICs of sampling locations at Oxford university libraries.

Sample TICs measured at each location (A, B and C) within the 7 sampling sites are given in Figures 2,3,4,5,6,7,8,9. Analyte concentrations, when measured by comparison with calibrant solutions, are given in Table 5. There was a wide range of VOCs present in the indoor air at all sampling locations whether paper-based materials were present or not.

**Table 5:** **Active sampling results for VOC collection / μg m**^**-3**^

	**NAS location**			**NLS location**			**BL location**			**CUL location**			**TNA location**			**TCD location**			**NLW location**			**OULS location**		
	**A**	**B**	**C**	**A**	**B**	**C**	**A**	**B**	**C**	**A**	**B**	**C**	**A**	**B**	**C**	**A**	**B**	**C**	**A**	**B**	**C**	**A**	**B**	**C**
**Heptane**	0.4	1.3	22	0.4	0.3	2.8	0.6	0.4	-	2.2	0.3	0.3	0.8	0.5	2.2	0.2	<0.1	0.1	0.9	0.2	<0.1	<0.1	<0.1	26
**Toluene**	1.9	1.0	103	1.9	1.2	1.6	1.7	2.2	0.9	72	6.1	68	4.5	2.6	1.7	1.2	0.9	0.6	0.5	0.2	1.7	1.6	1.1	1.3
**Furfural**	7.2	1.9	0.9	0.2	0.9	-	59	18	-	16	110	2.4	2.3	6.5	0.6	18	3.9	1.7	7.1	6.4	1.0	9.0	20	0.9
**Ethylbenzene**	0.1	0.1	1.3	0.2	0.2	0.3	1.0	0.5	0.2	4.7	0.4	0.4	0.8	0.4	0.4	0.3	0.1	0.1	0.2	0.1	0.4	1.4	0.3	0.2
**o-xylene**	0.7	0.6	6.8	0.7	0.6	1.3	1.1	2.0	1.0	25	1.6	1.0	1.6	1.3	1.1	1.2	<0.1	0.6	0.4	0.3	0.8	3.9	0.5	0.5
**p-xylene**	0.5	-	2.8	0.3	0.4	0.5	8.0	1.1	0.4	11	0.9	0.6	0.5	0.5	0.5	0.6	<0.1	0.2	0.6	<0.1	<0.1	2.7	0.5	0.3
**Cyclohexanone**	< 0.1	< 0.1	< 0.1	<0.1	< 0.1	<0.1	<0.1	<0.1	<0.1	<0.1	< 0.1	4.2	<0.1	<0.1	<0.1	0.6	<0.1	<0.1	<0.1	<0.1	<0.1	<0.1	<0.1	<0.1
**Nonane**	< 0.1	1.5	3.6	2.3	2.6	1.9	28	<0.1	<0.1	13	5.9	10	<0.1	0.5	<0.1	0.3	3.4	1.6	1.9	3.7	5.0	29	1.1	1.4
**4 ethyltoluene**	< 0.1	< 0.1	< 0.1	<0.1	0.2	0.3	15	<0.1	<0.1	13	15	<0.1	<0.1	0.5	<0.1	<0.1	<0.1	<0.1	0.2	-	1.2	<0.1	<0.1	<0.1
**Benzaldehyde**	2.0	2.2	2.5	0.8	1.0	1.7	4.5	3.4	0.6	54	15	6.7	0.7	2.1	1.6	1.7	0.5	<0.1	2.0	2.2	1.2	<0.1	2.9	1.6
**1,3,5 trimethylbenzene**	< 0.1	<0.1	<0.1	<0.1	0.1	0.1	7.2	0.2	-	8.3	5.2	<0.1	<0.1	0.2	0.2	0.4	<0.1	<0.1	<0.1	0.3	<0.1	<0.1	0.2	<0.1
**2 ethyltoluene**	< 0.1	< 0.1	< 0.1	<0.1	< 0.1	<0.1	4.9	<0.1	<0.1	5.3	<0.1	<0.1	<0.1	<0.1	<0.1	0.2	<0.1	<0.1	<0.1	<0.1	<0.1	5.8	<0.1	<0.1
**1,2,4, trimethylbenzene**	< 0.1	< 0.1	< 0.1	<0.1	< 0.1	0.9	23	<0.1	<0.1	38	23	<0.1	<0.1	<0.1	<0.1	2.5	1.2	<0.1	<0.1	<0.1	<0.1	10	2.6	<0.1
**Decane**	0.7	1.6	4.8	3.8	7.8	5.8	8.3	13	3.6	<0.1	32	66	1.4	<0.1	<0.1	0.7	16	3.7	9.6	23	12	73	6.6	5.1
**Limonene**	<0.1	2.3	4.7	0.6	1.0	34	<0.1	<0.1	5.3	<0.1	1.7	2.8	<0.1	<0.1	<0.1	0.5	0.5	<0.1	-	7.0	<0.1	<0.1	1.5	2.0
**Acetophenone**	<0.1	0.1	1.1	0.4	<0.1	0.8	<0.1	<0.1	<0.1	<0.1	1.0	<0.1	0.2	0.3	0.5	0.4	<0.1	<0.1	0.4	-	0.5	<0.1	<0.1	<0.1
**Camphor**	0.4	<0.1	<0.1	1.8	<0.1	<0.1	<0.1	<0.1	0.4	<0.1	0.2	<0.1	0.8	<0.1	<0.1	<0.1	<0.1	<0.1	<0.1	<0.1	<0.1	<0.1	<0.1	<0.1

At the NAS key volatiles detected in areas with paper-based collections (see Figure 2) included heptane, toluene, hexanal, furfural, isomers of xylene, heptanal, benzaldehyde, phenol, benzyl alcohol, nonanal and camphor (identifications confirmed by comparison to calibrant VOC standard solution). At the reference location, the most concentrated VOC analyte measured was toluene at 103 μg m^-3^ which was significantly higher than any other analyte detected. The cause of this spike (together with high levels of heptane, xylene and organic alcohols) was attributed to the use of the petroleum based adhesive Styccobond F60 which was applied to the flooring of the hallway during the sampling campaign. Although the contaminated sampling reference made it difficult to determine which pollutants, if any, arose due to emission of VOCs from the collection there were a few analytes, principally aldehydes, whose concentrations were significantly higher in locations A and B: hexanal, furfural, heptanal and nonanal. At the NLS (Figure 3) the TIC for location C was dominated by a peak for limonene (whose source is most likely a lemon-scented cleaning fluid) and a siloxane peak (due to column breakdown). The TICs representing analytes collected from shelves containing books are given relative to the reference TIC. Many analytes were present in the reference and the sampling TICs (heptane, toluene, ethylbenzene, xylenes, nonane, decane and nonanal). Examination of the BL data (Figure 4), indicated that the basement location surveyed (location A) was heavily contaminated with furfural (at approximately 59 μg m^-3^) and isomers of xylenes, ethyltoluene and trimethylbenzenes. The concentration of furfural was also high at the Colindale site (location B, at 18 μg m^-3^) compared to the reference sample C taken in an entrance hallway (0.03 μg m^-3^). A similar pattern was observed for paper-based sites at CUL (Figure 5); indeed the highest concentration of furfural measured in this study was at location B, Tower 14, Case 11 where a concentration of 110 μg m^-3^ was measured. The second sampling site, B, containing paper-based materials was also significantly higher for furfural concentration at 16 μg m^-3^ compared to the reference location C (2 μg m^-3^). High toluene spikes were observed for location A but also for the reference location C and so was not thought to have been generated as a result of sampling being conducted within a space that contained objects. In general, at the CUL, data generated in sampling sites with paper-based objects had higher concentrations of furfural, benzaldehyde, ethylbenzene, xylenes, and trimethylbenzene. Apart from the siloxane spike, at approximately 22.5 min, in the reference TIC for location C at TNA (Figure 6) similar concentrations of detected analytes were measured at all locations, except furfural whose concentration was higher at locations A and B. Significantly elevated concentrations of furfural were again detected at TCD sites A and B where paper-based objects were stored compared to the reference site (see Table 5 and Figure 7). Other analytes that were notably more concentrated at sampling sites A and B included the isomers of trimethylbenzene, nonanal and decane (location B). At the NLW (Figure 8), the collected TICs indicated that higher analyte concentrations were measured at the reference location apart from hexanal, furfural and trimethylbenzene which were higher for locations A and, or B. Finally at OULS (Figure 9) once again higher concentrations of furfural, decane, nonanal and trimethylbenzenes were measured at sample sites (locations A and B) where the collection was based.

In general the sampling survey indicated the presence of a large variety of VOCs present at all sampling locations including: heptane; toluene; ethylbenzene; o-xylene; p-xylene; nonane; benzaldehyde and decane. Other analytes (cyclohexanone, 4-ethyl toluene, 1,3,5-trimethyl benzene, 2-ethyl toluene, 1,2,4-trimethyl benzene, limonene acetophenone and camphor) were observed at some, but not all locations. Examination of the data in a univariate way indicated that furfural was the only analyte to be present consistently at significantly higher concentrations for sampling locations A or B, where paper-based materials were stored, compared to the reference location C. Measured concentrations in the collections ranged from 0.2 to 110 μg m^-3^, with a mean and standard deviation of 17.9 ± 28 μg m^-3^, whereas the measured concentration range over location C at all sites was 0 – 2.4 μg m^-3^ with a mean and standard deviation of 0.94 ± 0.81 μg m^-3^.

**Figure 10:**
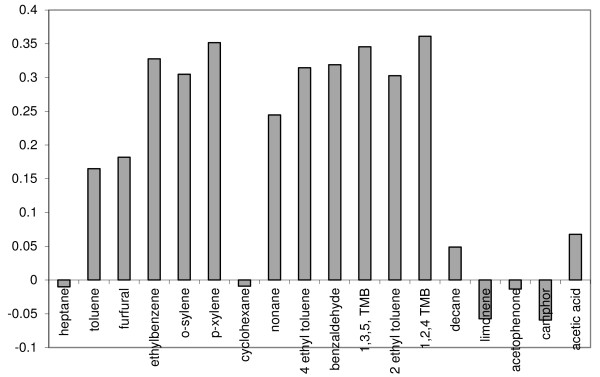
Loadings plot for PC1, indicating 40% of the variability in the data.

**Figure 11:**
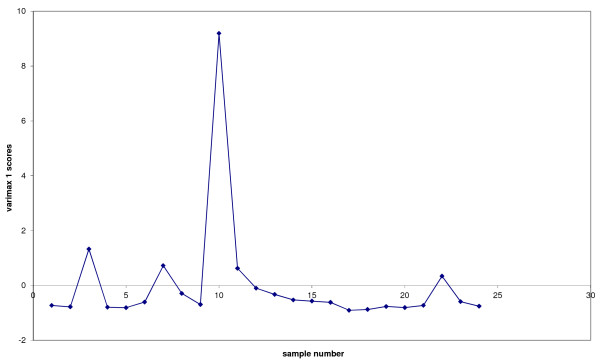
Scores plot for PC1, highlighting sampling site 10 (CUL location A).

**Figure 12:**
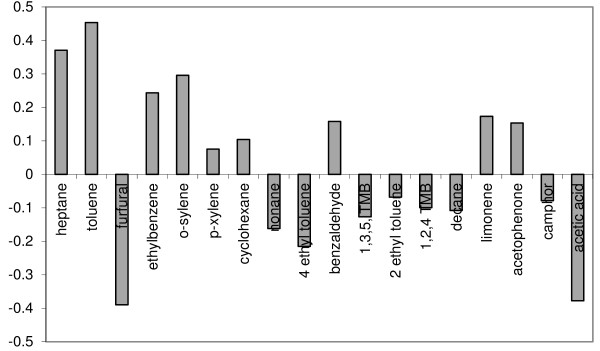
Loadings plot for PC2, indicating 40% of the variability in the data.

**Figure 13:**
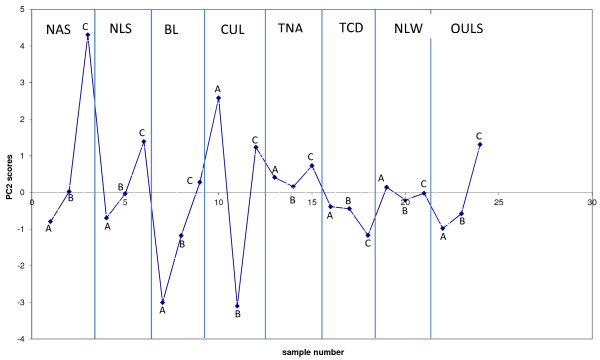
Scores plot for PC1, indicating a general high PC2 score for location C compared to locations A and B.

The analytical data in Table 5 and the acetic acid data were also examined by multivariate analysis using principal component analysis (PCA) to identify correlations between sampling locations or variables; the sampling locations (1 – 24) were entered in the same order as the columns listed in Table 5 and so every 3^rd^ point in the data refers to the reference location C. PCA analysis was used to produce loading and scores plots. Examination of loadings plots provide information on which variables (analytes) are correlated. For example analytes with a loading value of zero are not correlated with any other analyte. If two or more analytes have high loading values this implies that they are strongly associated and have similarly high concentrations. In contrast large negative loading values imply that analytes are still correlated but with relatively lower analyte concentration. Examination of the loadings plot for PC1 (Figure 10), which described 40% of the data, indicated a positive correlation between almost all measured analytes. This implied that it was not possible to distinguish between these analytes at the different sampling sites. This was also evident by examination of the scores plot for PC1 (see Figure 11) where most of the scores for each sampling site were close to zero implying that analyte combinations measured at each site were not correlated. (Note that sites demonstrating high, similar scores, would imply that they have similar concentrations of those analytes which are correlated with that principal component). Only 1 site had a high score value CUL location A indicating that on balance the measured concentrations of toluene, furfural, ethylbenzene, o-xylene, p-xylene, nonane, ethyltoluene, benzaldehyde, and the trimethylbenzenes were all similar regardless of sampling location. Perhaps of more interest was the loadings plot associated with PC2 (Figure 12) which illustrated a relatively strong correlation between heptane and toluene (they had the highest positive loading values), which were anti-correlated to furfural and acetic acid (the highest negative loading values). These four analytes are then be used to evaluate the scores plot for PC2 (Figure 13). When the PC2 scores plot was examined across all sampling locations there appeared to be a pattern in the data in that every 3rd point, the institutions’ chosen reference location C, was generally higher than the previous 2 points. In other words location C, normally had higher concentrations of heptane and toluene (high loading values for PC2), with lower concentrations of acetic acid, furfural, (high negative loading values for PC2). Indeed, only one score for PC2 had location C plotted below zero (sample number 18) which related to location C at Trinity College Dublin; likely due to the unusually high concentration of acetic acid measured there.

### Book sampling using SPME fibres or PDMS sampling strips

**Figure 14:**
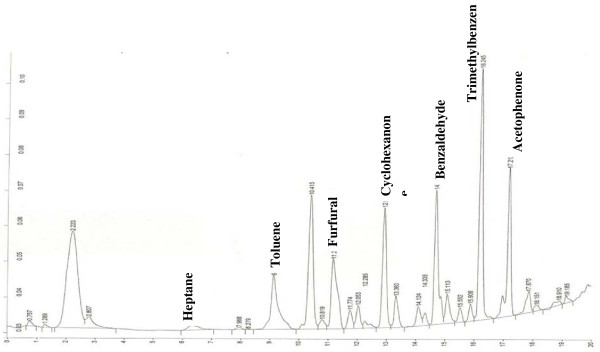
GC-FIC chromatogram of SPME fibre placed inside ‘The Determination of Hydrogen ions’, 1923.

Four books (see Table 2) were analysed by SPME fibre or PDMS strips. The SPME fibre was shown to collect a range of VOCs from the books, a typical GC-FID chromatogram is given in Figure 14. A number of peaks were identified by comparison of their retention time and peak shape with those of a standard solution. Of those measured by SPME emitting directly from paper within the book heptane, toluene, furfural, benzaldehyde and trimethylbenzene were 5 analytes that featured in the VOC sampling campaign indicating that their source may have been from the deterioration of paper. In addition cyclohexanone and acetophenone were also detected at significant concentration, although these analytes were not measured at significant concentration during the indoor air sampling campaign. Difficulties were encountered when attempting to quantify the concentrations of VOCs emitted from the books as information on their partition coefficients into the relevant stationary phase was not available, however if the peak areas were compared relative to each other the highest concentrations of VOCs obtained were collected from the book entitled ‘Determination of Hydrogen Ions’ from 1923. As a comparison, a new text book entitled ‘Analytical Chemistry published in 1997, was also assessed and no VOCs were measured after the same collection period.

**Figure 15:**
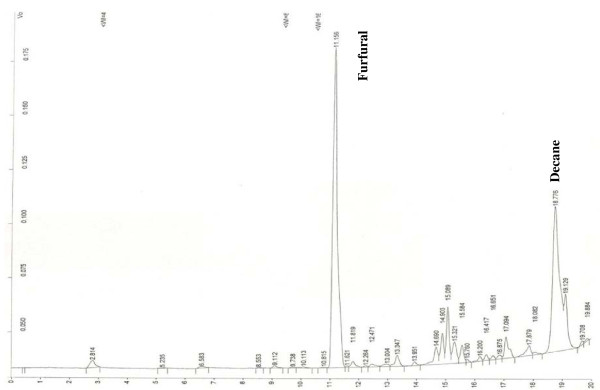
GC-FIC chromatogram of SPME fibre placed inside ‘Whitaker’s Almanack, 1903, stored at the British Library.

**Figure 16:**
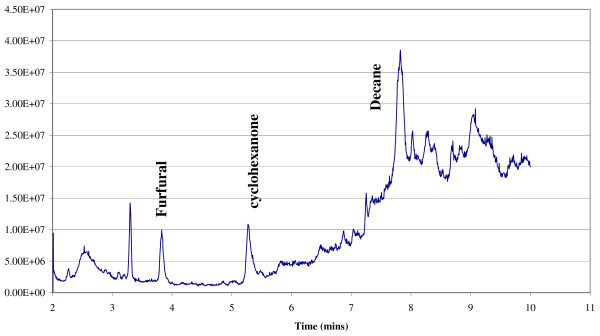
TIC of analysed PDMS strip placed inside Whitacker’s Alamnac, 1903 at the National Archives of Scotland.

Analyses of the SPME fibres that had been placed inside the Whitaker’s Almanack stored at each institution gave similar chromatograms with significant peaks of heptane, toluene, furfural, ethylbenzene, benzaldehyde, acetophenone and decane (see Table 6). What was of interest was that there did not appear to be any correlation between the trapped quantities of heptane, toluene, ethylbenzene or acetophenone. In contrast however, there was a strong correlation between furfural and decane emitted from each Whittaker’s Almanack perhaps indicating that the levels of these 2 analytes in combination may provide some information about the stability of paper (see Figure 15 which illustrates the highest levels of furfural and decane measured from the book stored at the British Library during the SPME analyses). The same Whitacker’s Almanacks were analysed using PDMS strips and the same VOCs were detected however this method of collection was less sensitive (as expected due to a much lower surface area and lack of stationary phase). However, it was still possible to identify furfural, cyclohexanone and decane in each TIC (see Figure 16 as an example).

**Table 6:** Results (integrated peak areas) of SPME analyses of Whitacker’s Almanack (1903)

**Analyte**	**NAS**	**NLS**	**BL**	**CUL**	**TCD**	**NLW**	**OULS**
Heptane	1740	3633	1130	2879	531	2250	2828
Toluene	3505	27923	1159	2001	1551	2971	6034
Furfural	50864	87600	**165629**	81534	73890	63607	133581
Ethylbenzene	3748	3582	< 1000	< 1000	< 1000	4469	6264
Benzaldehyde	9304	4842	< 1000	16513	< 1000	< 1000	12704
Acetophenone	6223	8467	10139	< 1000	3041	5624	< 1000
Decane	42112	69419	**109054**	22827	12224	24186	56537

Three books (listed in Table 3) were also analysed using PDMS strips and the mass of furfural trapped was determined by calibration. The mass of furfural correlated with the age of the sampled book with average masses of < 0.10, 0.65 and 16 ng trapped by the PDMS strip per day for the books that were produced in 2003, 1988 and 1952, respectively. Moreover the mass of furfural trapped increased with sampling time. To assess whether the mass of furfural varied across the page of a book, PDMS strips were placed inside the book produced in 1952. Elastomer strips were placed either at the edge of the open face of the book or in the centre. After a collection time of 3 days an average mass of 16 ng of furfural was collected at the edges of the page whereas an average mass of 5 ng of furfural was collected at the centre of the page, suggesting that higher emissions (and subsequently paper deterioration) will occur on paper held at the edges of a book compared to the centre.

**Figure 17:**
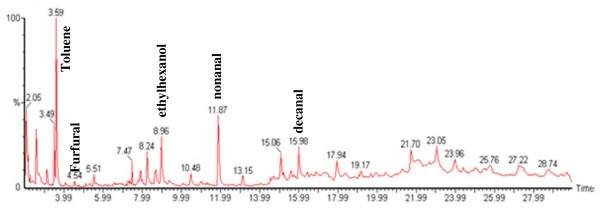
TIC of analysed PDMS strip placed inside 19th c book and binding at the University of Glasgow Archives.

To test the use of PDMS strips in books for use over a longer period of time (to increase the sensitivity of the collection method), a number of books were selected for study at the Glasgow University Archives (see Table 7). Interestingly the same analytes were detected in some samples by the PDMS strips; toluene, furfural, benzaldehyde, nonanal and decanal, although they were trapped at much higher concentration after the paper was exposed to the PDMS collection strip for a longer period of time (Figure 17); indicating that even though this study reports the preliminary use of PDMS strips as VOC collection devices, the data presented here illustrates their potential use as a non-invasive tool to assess paper and its stability.

**Table 7:** **Analyte masses (ng cm**^**-2**^**) trapped on books held at Glasgow University Archives**

**Date of book sampled**	**Toluene**	**Furfural**	**Benzaldehyde**	**ethylhexanol**	**Nonanal**	**Decanal**	**Thymol**
Cm. 1.5: Rome, 1470.	3.8	< 0.01	0.20	0.72	0.55	2.4	< 0.01
Bx. 3.25: incunables with washed out annotations; 1471	1.8	0.20	0.42	1.1	1.7	1.4	1.5
SM1984&BD9-d.13: 2 similar bindings; 1481	6.1	0.34	0.49	2.6	1.5	1.1	< 0.01
Poliziano, Angelo,. 1498.	6.3	0.36	0.51	0.97	5.4	0.70	< 0.01
Book covers from 1498.	3.8	0.42	0.39	0.67	0.97	0.33	< 0.01
Bv. 2.31: The chronicles of England, 1480	< 0.01	< 0.01	< 0.01	< 0.01	< 0.01	< 0.01	1440
17^th^ c work and pasteboard type binding; Leipzig, 1694.	7.4	0.44	0.58	2.8	1.9	1.5	0.51
Aq-e 23: 18^th^ c work on browing paper; London 1721.	4.0	0.37	0.39	1.3	0.66	1.2	1.6
RB 4765: 18^th^ c pamphlet; 1739-1767	1.5	0.22	1.0	1.5	1.2	3.3	< 0.01
Z2-d.6: 19^th^ c work; 1835.	3.7	0.55	0.36	1.7	3.5	1.4	< 0.01
RQ:3071: 19^th^ c book and binding: 1810,1811	3.4	0.45	0.33	1.8	3.0	1.3	< 0.01
RQ 3052: 29 19^th^ c pamphlets in modern cloth binding.	3.6	1.6	0.47	2.3	4.8	3.0	< 0.01
Early / mid 19^th^ c loose letters (incoming and copy letters)	2.8	0.37	0.31	1.3	0.92	0.90	< 0.01
Late 19^th^ c letterbook:	8.4	3.5	0.30	1.0	1.6	0.84	< 0.01
1940s loose papers (mainly typed duplicates):	6.6	0.73	0.35	1.5	2.4	1.1	< 0.01
1950 photostats in volume: MS Farmer 702	8.7	0.15	0.16	1.32	1.7	0.47	< 0.01
Trotsy periodicals:acidified newspaper from 1946 (US).	0.57	< 0.01	< 0.01	< 0.01	< 0.01	0.73	< 0.01
Modern binding in a box 19^th^ c.	4.0	0.72	0.86	2.4	2.4	1.4	< 0.01
Late 20^th^ c ms notes/offprints: MS Hobsbaum (Ben Johnson)	2.9	0.85	0.83	2.6	2.9	1.0	< 0.01

## Experimental

### Materials

All chemicals were supplied by Sigma-Aldrich, Dorset, U.K. and used without modification: sodium tetraborate, methanol, ethanol, acetonitrile, Tenax TA, sodium acetate, sodium formate, heptane, furfural, toluene, ethylbenzene, o-xylene, p-xylene, nonane, cyclohexanone, 1,2,3 trimethylbenzene, 1,2,4 trimethylbenzene, 1,3,5 trimethylbenzene, 2-ethyltoluene, 3-ethyltoluene, 4-ethyltoluene, decane, acetophenone, camphor, limonene, formaldehyde-dinitrophenylhydrazine and orthophosphoric acid. 2, 4-dinitrophenyl-hydrazine (2,4-DNPH) was supplied by Mallinckrodt Baker and was doubly recrystallised in ethanol before use. Sheets (300 mm × 300 mm × 6 mm) of silicone elastomer (polydimethylsiloxane, PDMS) were supplied by GoodFellow and SPME fibres were supplied by Supelco.

### Passive sampling

Palmes diffusion tubes, (supplied by Gradko International U.K.), were used to examine the concentration of acetic acid, formic acid, formaldehyde and sulfur dioxide at each location. The sulfur dioxide tubes were prepared and analysed by Gradko International whereas the organic acid and aldehyde tubes were prepared and analysed at the University of Strathclyde using previously published methods [20,21]. Two sampling tubes of each type were placed side-by-side at the chosen sample location for 28 days. Prepared sampling tubes contained a large excess of trapping reagent so that < 5% of the reagent was derivatised during sampling. This ensured that sampling rates were not hindered during the full sampling period. Moreover, as the pollutants were trapped and derivatised by chemical reagents to non-volatile products, there was no opportunity for back-diffusion during sampling.

### Active sampling method

Thermal desorption tubes filled with Tenax TA (supplied by Perkin-Elmer, U.K.) were used to actively sample sites for volatile organic compounds (VOCs). Each sampling tube was conditioned prior to use in a thermal desorption unit (Perkin-Elmer Turbo Matrix TD) at 320°C for a period of 20 min and, after conditioning, sampling tubes were sealed using brass caps before being shipped to each sampling location. When on-site, air was pulled through each sampling tube using an SKC Universal sampling pump after the flow rate through the sampling tube was calibrated to 100 cm^3^ min^-1^ using a bubble meter. At each site, the environment was sampled for a total period of 24 h giving a sampling volume of approximately 144 dm^3^.

A Perkin-Elmer Turbo Matrix TD instrument coupled to a Perkin-Elmer Autosystem XL gas chromatograph (GC) and Turbo Mass Gold Mass Spectrometer (MS) was used to thermally desorb the VOCs from the active sampling tubes. The analytes were desorbed from the tube held at 300°C for 5 min. Released volatiles were focussed onto a Tenax TA cold trap held at −30°C. The second stage of the desorption process involved a rapid heat of the cold trap to 300°C (99°C s^-1^) for 5 min. Desorption and inlet splits were combined to dilute the desorbed analytes and a 0.6% volume of eluate passed into the GC-MS using a heated transfer line at 250°C.

The analytes were separated on a Perkin-Elmer, SMS Elite, (dimethylpolysiloxane 5% diphenyl) column, 30 m × 0.25 mm i.d using He at 1 cm^3^ min^-1^. The GC oven temperature was programmed as follows: an initial temperature of 35°C for 10 min, increased to 175°C at 5°C min^-1^ and held at 175°C for 2 min. The mass spectrometer was set to scan the m/z range of 50–300 for a period of 40 min with a scan time of 0.2 s. The multiplier was set at 350 V with an electron energy of 70 eV.

### Analysis of SPME fibres and PDMS strips

VOCs trapped onto SPME fibres were analysed using GC with a 5% phenyl column held at 35°C for 5 min before a ramp of 7.5°C per min was used until 200°C. The injection port and flame ionisation detector were held at 220 or 250°C, respectively. To identify trapped volatiles the PDMS strips were placed inside empty stainless steel sampling tubes (Perkin Elmer) and analysed using the same TD-GC-MS conditions as outlined above. When using thermal desorption to analyse PDMS strips a maximum desorption temperature of 110°C is strongly recommended otherwise a range of siloxane breakdown products is identified in the resultant total ion chromatograms (TICs).

## Conclusion

At the locations surveyed here, it was shown that high concentrations of a wide range of VOCs were measured in the indoor air of selected locations at the national libraries and archives. What the VOC signature means is open to debate and care needs to be taken to link the presence of VOCs with cellulose degradation. Many VOC compounds were measured in storage rooms with a large volume of paper based collections, however, some analytes were also measured in reference locations in the absence of objects. Based on the results given here it is suggested that elevated concentrations of a combination of analytes may indicate emissive products produced as a result of paper deterioration; acetic acid, furfural, ethyltolune, trimethylbenzenes, decane and camphor. Examination of analyte concentrations in combination might be of use to indicate a degree of cellulose breakdown in the collection. These analytes were consistently higher in concentration when paper-based materials were stored, compared to corridors or entrance hallways acting as sampling reference measurements. Care should be taken not to use univariate analyte concentration assessment as VOC concentrations do vary considerably.

Emissive analytes can also be directly sampled from paper in books using SPME fibres or PDMS strips. The SPME fibres are more sensitive and will provide more information in a shorter sampling period, however, during the course of this work, 2 of the 7 SPME fibres were damaged when placed inside a book. Perhaps a reasonable alternative to direct contact SPME fibre sampling would be the use of PDMS strips. Each strip costs approximately £0.30, compared to a SPME fibre at approximately £90, and can be easily slipped within the pages of a book to start collecting emissions. To increase the concentration of analyte measured the strips could be placed inside the books for longer periods of time. Moreover, as a number of PDMS strips can be placed inside the pages of a book simultaneously, based on the preliminary results given here, it is suggested that they can be used to provide spatial distribution of emissions within a book to determine whether deterioration occurs more rapidly at the edges or at the centre of a book. However, more work is required to correlate the detected analytes with paper of varying, and known, stability ranges before they can be used as inferential measurement devices for paper deterioration.

## Competing interests

The authors declare that they have no competing interests.

## Authors’ contributions

LTG: design of experiments, conduct analysis, interpret results and write paper. AEA: analysis and data interpretation of PDMS strips from Glasgow University Archives. BK: Data interpretation and article review. VH: Data interpretation and article review. GM: Analysis of SPME and PDMS fibres in books. CJR: Indoor air survey of library and archives. All authors read and approved the final manuscript.
